# LBMR: Load-Balanced Multipath Routing for Wireless Data-Intensive Transmission in Real-Time Medical Monitoring

**DOI:** 10.3390/ijerph13060547

**Published:** 2016-05-31

**Authors:** Chinyang Henry Tseng

**Affiliations:** Computer Science and Information Engineering, National Taipei University, New Taipei 237, Taiwan; tsengcyt@gm.ntpu.edu.tw; Tel.: +886-2-8674-1111 (ext. 67124)

**Keywords:** medical monitoring, multipath routing, load balancing, load estimation, route maintenance

## Abstract

In wireless networks, low-power Zigbee is an excellent network solution for wireless medical monitoring systems. Medical monitoring generally involves transmission of a large amount of data and easily causes bottleneck problems. Although Zigbee’s AODV mesh routing provides extensible multi-hop data transmission to extend network coverage, it originally does not, and needs to support some form of load balancing mechanism to avoid bottlenecks. To guarantee a more reliable multi-hop data transmission for life-critical medical applications, we have developed a multipath solution, called Load-Balanced Multipath Routing (LBMR) to replace Zigbee’s routing mechanism. LBMR consists of three main parts: Layer Routing Construction (LRC), a Load Estimation Algorithm (LEA), and a Route Maintenance (RM) mechanism. LRC assigns nodes into different layers based on the node’s distance to the medical data gateway. Nodes can have multiple next-hops delivering medical data toward the gateway. All neighboring layer-nodes exchange flow information containing current load, which is the used by the LEA to estimate future load of next-hops to the gateway. With LBMR, nodes can choose the neighbors with the least load as the next-hops and thus can achieve load balancing and avoid bottlenecks. Furthermore, RM can detect route failures in real-time and perform route redirection to ensure routing robustness. Since LRC and LEA prevent bottlenecks while RM ensures routing fault tolerance, LBMR provides a highly reliable routing service for medical monitoring. To evaluate these accomplishments, we compare LBMR with Zigbee’s AODV and another multipath protocol, AOMDV. The simulation results demonstrate LBMR achieves better load balancing, less unreachable nodes, and better packet delivery ratio than either AODV or AOMDV.

## 1. Introduction

Recently, the demands of remote medical monitoring [1] keep growing. WiFi and Bluetooth are the most popular wireless media for wireless remote monitoring. For example, Thelen *et al.* used Bluetooth for emergency medical services [2]. To overcome the disadvantage of Bluetooth’s limited coverage, a WiFi-based medical monitor system was developed [3]. With the increasing popularity of smart phones, the design of medical monitoring systems on Android mobile terminal equipment is also proposed [4]. Furthermore, a wearable health monitoring sensor integrated with a body-area network [5] is also gaining market attention.

Patients are sensitive to high radio transmission power devices supported by WiFI, whose transmission power is usually 100 mW [6]. Compared with WiFi, Zigbee’s low transmission power (1 mW) [6] constitutes a healthier network solution for medical sensor devices, which usually have a short distance to the human body and require low radio emissions [7]. To overcome the short wireless coverage problem, the Zigbee stack [8] supports mesh routing by adopting the so-called Ad hoc On-demand Distance Vector (AODV) [9] method to automatically construct an *ad hoc* network as routes are needed. To collect medical data through wireless transmissions from long-distance remote sites, AODV mesh routing can extend sensor coverage with multi-hop transmission. Since Zigbee has advantages regarding body-health and remote-transmission aspects, numerous Zigbee-based medical monitoring systems have been developed [10,11,12], allowing patients to maintain their independence and mobility, but without addressing potential bottleneck problems.

What is *“bottleneck”?* In general, medical monitoring systems such as electrocardiography (ECG), with its continuous sample transfer, involve transmission of large amounts of data, and can easily cause a point of transmission failure when nodes become jammed with excessive data which cannot be processed in time, then bottlenecks happen. In Zigbee, during the process of data aggregation towards a data collection gateway, nodes near the gateway may experience more network traffic than others and thus become bottlenecks and cause unreliable data delivery, such as packet drops due to the network congestion at the bottlenecks. To solve bottlenecks, load balancing is critical for avoiding data congestion at pre-known busy nodes, especially for data-intensive applications. However, the design of Zigbee’s AODV routing, which has been used for numerous medical monitoring systems [10,11,12], only focuses on optimizing routing paths to be the shortest without seriously considering load balancing issues. In addition, bottlenecks may cause broken links to trigger AODV Route Error (RERR) messages. Once RERRs are generated, nodes receiving RERR messages subsequently generate more REER messages. Eventually, the enormous volume of AODV RERR messages can paralyze the entire network, especially the links close to the gateway. Therefore, as the network demands of medical monitoring applications grow, Zigbee’s routing service can become very unreliable. This is the problem our research intends to solve.

To avoid bottlenecks, many load-balance wireless routing systems have been proposed. Pure cluster solutions [13,14] aim at building as uniformly small-sized clusters as possible to achieve cluster load-balancing. Advanced cluster solutions [15,16] balance energy consumption on each node and indirectly achieve load balancing. Liao [15] built a balanced clustering structure and thus avoided forming large clusters. In each cluster, the node with the highest energy is elected as the cluster head for aggregating inter-cluster data to the base station. Wu [16] proposed a centralized power efficient routing algorithm to determine cluster heads and build a multi-hop routing protocol among all cluster heads. ME-AODV [17] adds the *multipath* concept to energy-aware load-balancing solutions. ME-AODV uses multiple paths in a round robin fashion to evenly distribute energy consumption over the entire network. The Neighbor-aware Adaptive Load Balancing algorithm [18] uses the information of parent and child nodes along with a probability factor to balance the traffic and prolong network lifetime. All nodes send traffic load information to the gateway which subsequently calculates and broadcasts the determined probability factor used by the whole network. 

In order to provide a reliable routing service for data-intensive medical applications, we propose Load-Balanced Multipath Routing (LBMR). When designing LBMR, we prefer a non-cluster architecture because cluster heads potentially cause more bottleneck candidates. In data-intensive medical monitoring, reliable data transmission becomes more critical than saving energy. Therefore, LBMR takes traffic load as the cost function and adaptively updates load information with neighbors to calculate the least busy routes. Besides, LBMR uses multipath routing to provide more reliability. The main comparison with LBMR is Ad Hoc On-demand Multipath Distance Vector (AOMDV) [19]. AOMDV is a multipath solution based on AODV that can transmit data through equivalent paths with the same hop-counts to the gateway. The main purpose of the AOMDV multipath method is to enhance network reliability and avoid failure points. Obviously, the characteristics of the AOMDV multipath method have potential to improve load balancing for AODV. Unfortunately, AOMDV’s equivalent paths must be thoroughly disjoint and cannot share nodes on their distinct paths. This limits the number of available alternatives. Besides, AOMDV does not consider traffic load when selecting the sending path and cannot maximize load-balancing effect. In addition, AODV and AOMDV are designed for mobile *ad hoc* networks so their routing designs are not optimized for data-intensive services.

To develop a better multipath solution to support data-intensive medical monitoring services, LBMR has a reliable layering architecture, where the routing computation is done locally among neighbors. This distributed layer architecture can avoid another bottleneck problem at cluster heads, such as the coordinator node in a Zigbee network. Besides, LBMR utilizes traffic load as the cost function and solves imbalanced load problems more directly than AOMDV. Furthermore, LBMR can enhance network reliability by providing multiple next-hop options and guarantees the shortest paths are selected. 

LBMR consists of three main designs: Layer Routing Construction (LRC), Load Estimation Algorithm (LEA), and Route Maintenance (RM) mechanism. In LRC, the data gateway is the top level and we define that nodes *closer* to the gateway are in the *upper* layers and nodes *farther* from the gateway are in the *lower* layers. Each sensor node may play both lower-layer and upper-layer node roles depending on the relative distance to the gateway compared with their neighbors. Each lower-layer node only needs to know the *local* information of next-hop nodes in the immediate upper layer, and the path from source to the gateway is constructed hop-by-hop. This structured and inductive two-layer relationship establishes the reliable routing service. Furthermore, LEA allows each lower-layer node to calculate which upper-layer node has the least traffic load. Consequently, the upper-layer node with less traffic is selected as the next-hop to the gateway. Through the cooperation of LRC and LEA, local load-balancing optimization is accomplished. In the LBMR routing table, multiple paths are recorded and this allows more fault-tolerance once some next hop fails. Furthermore, another assurance of delivery quality is RM. Once any node fails to operate normally, RM allows lower-layer nodes to recalculate their best upper-layer nodes toward the gateway without broadcasting route error messages. Therefore, LBMR can quickly adapt sensor nodes to dynamic flow changes and malfunctioning links.

The main contributions of LBMR are multipath routing with load balancing, robustness, and reliability. First, load balancing is done by selecting the best upper-layer node with the least traffic load. Second, robustness is achieved because the synergy of LRC and LEA provides multiple upper-layer next-hops to the gateway for each lower-layer nodes, and RM can detect link failures for quick link switching. Since LBMR eliminates bottlenecks by load balancing design and provides multipath routing, LBMR provides a much more reliable routing service than current Zigbee AODV-related solutions. Simulation results show LBMR provides better load balancing and achieves better route maintenance with more reliable packet delivery comparing with AODV and AOMDV. 

The rest of this paper is organized as follows: Section 2 illustrates how Zigbee-LBMR assists wireless medical monitoring. Section 3 presents LBMR. Section 4 demonstrates our simulation results. Finally, Section 5 concludes this paper.

## 2. Scenario Illustration

**Figure 1:**
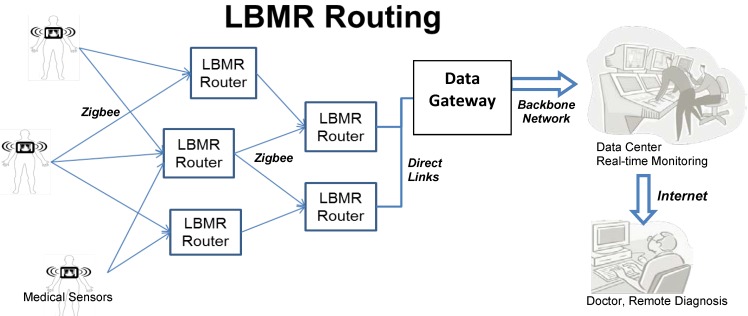
Example scenario of LBMR routing for real-time medical monitoring.

In Figure 1, patients carry wearable sensors collecting sensed medical data. Subsequently, medical data is transmitted through Zigbee with LBMR routing, which is what this paper focuses on, to reliably deliver a data-intensive wireless flow for real-time medical monitoring. LBMR can offer medical monitoring service a healthy, high-data rate, large coverage and robust transmission with balanced flow on distributed nodes. After the data safely and correctly arrives at the data center, professionals and doctors can perform remote diagnoses and provide timely warnings or advice. 

LBMR is proposed to cooperate with a large-scale wireless Zigbee network, in which two types of bottlenecks can occur. In a Zigbee Personal Area Network, medical data is usually sent to the coordinator, which manages the network and has direct links (*i.e.*, USB connections) to the gateway, and clearly the coordinator is the bottleneck of the network. Tseng proposed a successful remote ECG monitoring system of large scale Zigbee sensors [20]. This system eliminates this bottleneck with the Coordinator Traffic Diffusion (CTD) method to redirect excessive traffic from coordinator to the gateway. However, although CTD can diffuse the traffic near the coordinator, so the traffic load may not equally go through routers, when the traffic load increases significantly due to a large number of medical sensors, routers having more neighbors can experience relatively heavy traffic load and become a second type of bottlenecks. 

To prevent all possible bottlenecks, LBMR guides Zigbee routers in choosing next-hops with the lowest traffic loads. Each LBMR router in a Zigbee network serves network traffic equally to prevent the router from becoming a potential bottleneck. LBMR not only removes the bottlenecks near the gateway, but also eliminates possible bottlenecks in the entire Zigbee network. Therefore, LBMR routing ensures a reliable large scale Zigbee network service for data-intensive medical monitoring.

**Figure 2:**
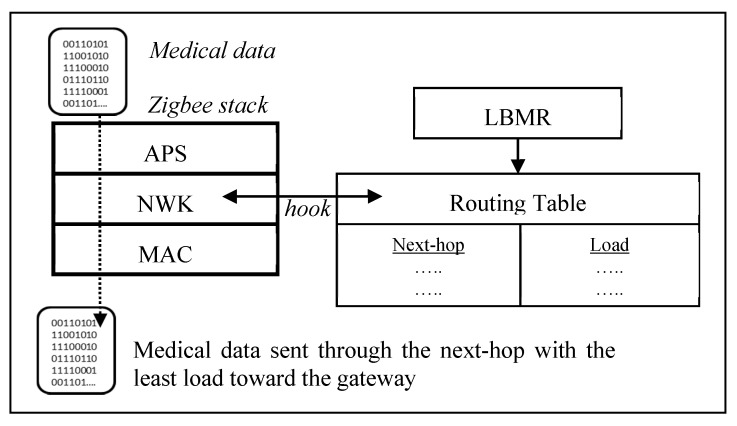
LBMR routing in a Zigbee stack.

Taking a closer look at LBMR in Figure 2, LBMR provides an alternative routing service for Zigbee networks without modifying the existing Zigbee stack by adding a hook at the network (NWK) layer, as shown in Figure 2. Once a Zigbee router forwards medical data, LBMR guides the Zigbee NWK layer to choose a next-hop with the least load toward the gateway. Besides, LBMR also ensures the next-hop is closer to the gateway than the router itself to prevent routing loops. Therefore, LBMR design can easily cooperate with existing Zigbee stacks.

## 3. LBMR: Load Balancing Multipath Routing

As medical applications grow rapidly, medical sensors may deliver intensive and critical data to the gateway so reliable medical routing service is highly desirable. Current solutions, such as Zigbee and related works mentioned in Section 1, cannot avoid bottlenecks, which may paralyze the entire network if the network traffic grows and congestion occurs near the gateway. To solve this problem, we propose LBMR to provide a reliable routing service for medical applications, especially data-intensive applications. Compared with AODV’s improved multipath version, AOMDV, LBMR has the same advantage of multipath routing but has better traffic load distribution and network reliability. In order to achieve load balancing, routing robustness, and routing reliability, LBMR consists of three main designs: LRC, LEA, and RM mechanism. LBMR is introduced from these three aspects in the following. 

In AODV and AOMDV, nodes require flooding RREQ to the gateway and receiving RREP from the gateway, which is usually several hops away from nodes. In LRC, nodes can simply receive the beacons from the upper-layer nodes, which are neighbors of the nodes, to establish the routes to the gateway, so the route establishment time is shorter in LMBR than the time in AODV and AOMDV. Based on LEA, LMBR accomplishes the load balancing service, which AODV and AOMDV does not provide. In AODV and AOMDV, nodes broadcast RERR to recover routes and this mechanism generates heavy message overhead and recovery time. RM in LMBR reuses beacons in LRC to announce and recover the failed routes. The recovery process in LMBR is very quick and requires no message overhead. Therefore, LMBR provides more reliable routing service with additional loading balancing feature than AOMDV and AODV, which are adapted by the Zigbee stack.

### 3.1. Layer Routing Construction 

**Figure 3:**
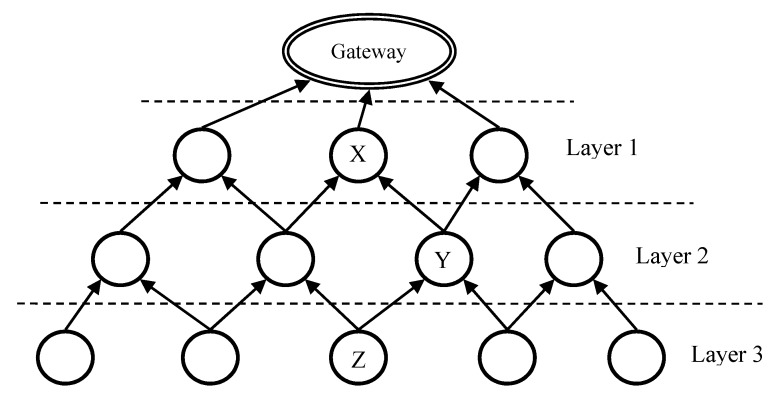
Layer routing in LBMR.

As shown in Figure 3, LRC is based on layer routing. The data gateway is the top level of the network. Each node, functions as a router, has a *layer value* indicating the distance to the gateway. *N^th^-layer* nodes are at the layer *N-hop* to the gateway. When the distance to the gateway is shorter, the layer value is smaller and the layer is upper, and *vice versa*. In LRC, nodes in the upper layers are closer to the gateway than nodes in the lower layers. Each sensor may play both roles of lower-layer and upper-layer nodes in different relationships. For example, the distances to the gateway of three nodes are denoted as X_d_, Y_d_ and Z_d_. Given that X_d_ < Y_d_ < Z_d_, Y is in X’s lower layer and Y is in Z’s upper layer. 

Therefore, there are several layering sets of LRC. Each lower-layer node only needs to know the information of next-hop nodes in the immediate upper layer to the gateway. In Figure 3, Layer-1 nodes are connected with the gateway directly through wired connections. All the other nodes communicate with one another through wireless connection. LRC can quickly build routes because it is a top-down approach and nodes only require partial-topology information for route construction. LRC provides available multiple paths with equivalent hops to the gateway and all these equivalent paths are the shortest ones. If one node fails, another close-by node can be another next-hop to the gateway. This reinforces the network robustness.

To initiate the network, all nodes including the gateway initialize their own layer values. The gateway sets its own layer value as 0 and all the other nodes set their initial layer value as 255. Subsequently, the gateway broadcasts a Route Construct message consisting of two fields, *Source Address* and *Layer Value*. When sensor nodes receive Route Construct messages, they take route construction action differently in three diverse conditions described in the next paragraph and the constructed routes are totally loop-free and the shortest paths to the gateway. 

Once a node X receives a Route Construct message from node Y, X checks Y’s layer value carried in the received Route Construct message. As illustrated in Table 1, X performs route construction according to different layer value conditions:

*Condition 1 (X’s layer value − 1 < Y’s layer value)*:

X discards this Route Construct message without adding Y to Routing Table because Y is farther from the gateway than X is. This guarantees a *loop-free* network.

*Condition 2 (X’s layer value − 1 > Y’s layer value)*:

X deletes all entries of its own Routing Table because Y becomes the best next-hop candidate. 

X updates its own layer value to (Y’s layer value + 1)

X adds Y to Routing Table.

*Condition 3 (X’s layer value − 1 = Y’s layer value)*:

X adds Y to its own Routing Table as another equivalent path to the gateway. Multiple next-hop choices can enhance routing robustness.

**Figure 4:**
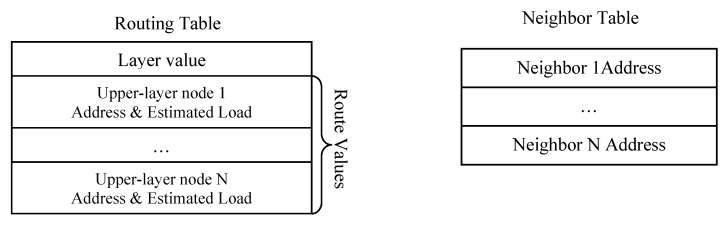
Routing table and neighbor table in LBMR.

The routing table of each node consists of *Layer value* and *Route values*, which has several upper-layer nodes associated with estimated loads. A next hop represents an upper-layer node on the path to the gateway, and estimated load is illustrated in LEA section in the following. Besides Routing Table, another Neighbor Table is used to record available neighbors to the gateway. Routing Table only stores the upper-layer nodes to the gateway, while Neighbor Table stores addresses of all neighboring nodes. Figure 4 shows the fields in Routing Table and Neighbor Table.

### 3.2. Load Estimation Algorithm

LEA is the core of LBMR. In order to achieve load balancing, every node periodically sends a Load Estimation message to notify neighbors of its own current traffic load. LRC allows nodes to have alternative paths to the gateway, and therefore nodes can choose the path with the least traffic as the next-hop to the gateway. If every sensor node always chooses the most unoccupied link, the network can become more load-balanced and bottleneck problem can be eliminated in the case where alternative routes are available. 

Each node periodically broadcasts Load Estimation message consisting of four fields: *Source Address, Estimated Load, Layer Value,* and *Routing Flag*. Routing flag is used for route maintenance and will be explained in the RM section. When receiving Load Estimation messages, nodes update their routing tables by adding new upper-layer nodes and the corresponding estimated loads. Once a node N receives a load estimation message M, it first compares M’s layer value and the layer value of upper-layer nodes in the routing table. If M’s layer value is smaller, node N updates the layer value in its routing table and subsequently replaces current upper-layer nodes with M’s source address with M’s estimated load. Otherwise, node N discards message M because a larger layer value implies a longer distance from the gateway. LEA uses an exponential weighted moving average (EWMA) formula [21] to calculate estimated load. EWMA can be used to predict future traffic and reduce fluctuation of future traffic flow. LEA estimates future load, which can reflect dramatic traffic changes in advance and avoid inaccurate next-hop selections.

The sample code of LEA is presented in Table 2. In LEA, i is time slot number, Estimated_Load_i_ represents the estimated load in the i^th^ time slot. Estimated_Load_i−1_ represents the estimated load of the (i − 1)^th^ time slot, which is the previous time slot of the i^th^ time slot. SampleLoad represents the current load and α is the parameter used to estimate future estimated load from SampleLoad.

In the beginning, Estimated_Load_0_ is set to SampleLoad. There are two conditions of SampleLoad for determining Estimated_Load_i_ in the i^th^ time slot. If SampleLoad is 0, the Estimated_Load_i_ is not set to 0 but to half of the previous estimated load to prevent the estimated load from dropping dramatically. If SampleLoad is non-zero, estimated load is determined by EWMA. α is the weight of SampleLoad and is used to determine the influence of current traffic load on predicting the future traffic load. The impact of α is discussed in Section 4 through our experiments. If the estimated load of some particular node N is larger, it means node N would potentially experience heavier traffic, and *vice versa*. In LEA, every node periodically calculates its own estimated load and broadcasts to neighbors through load estimation messages. While all nodes know the future estimated load of their upper-layer nodes, they can select the one with the least estimated load. Under this rule, the traffic is directed to the less busy channel and the network is well-balanced.

### 3.3. Route Maintenance Mechanism

RM is used to react to broken links. The network topology of medical monitoring applications may change due to node failure or newly joining nodes. Once any node fails to operate normally, RM can quickly adapt sensor nodes to dynamic flow change and mal-function links locally. As mentioned above, a load estimation message carries a routing flag, which is used to confirm whether the load estimation sender S has routes to the gateway or not. When the routing flag is 1, it indicates that S has at least one route in the routing table to the gateway. If S has no route to the gateway, Routing Flag is set 0 to inform others not to use S as the upper-layer node towards the gateway. Then RM mechanism allows lower-layer neighbors of node S to recalculate their best upper-layer nodes toward the gateway without broadcasting route error messages. Suppose node Y does not receive a load estimation from upper-layer neighbor X for a period of time, Y will remove X from both its neighbor table and routing table. If Y’s routing table has no upper-layer nodes to the gateway after removing X, the following actions start:

(1) Y will set routing flag to be 0 and layer value to be 255 and notice its neighbors by its next load estimation message.
(2) Y’s lower-layer neighbor Z removes Y from its own routing table and ignores Y’s new load estimation messages.
(3) From neighbors’ load estimation messages, Y adds neighbors with the lowest layer value as upper-layer nodes into its routing table and thus Y can be connected to the gateway again.
(4) Once Y has new upper-level nodes to the gateway, Y sets its own routing flag back to 1. Y can be added to others’ routing table, and its load estimation is considered by others again.

The beauty of LBMR is that each node only updates its own status in load estimation messages. LBMR nodes periodically check load estimation for each surrounding node’s status and react to link changes quickly. Once some particular LBMR node fails, no RERR messages, designed in AODV and AOMDV, are sent. Extra RERR messages increase routing overhead and link recovery time.

## 4. Experiments

This section evaluates LBMR compared with AOMDV and AODV, especially that AODV is used by numerous Zigbee based medical monitoring systems [10,11,12,20]. Evaluation metrics include load balancing, unreachable nodes, and packet delivery ratio. We use ns2 as the simulation platform, whose environment parameters are listed in Table 3. 

In the simulation topology, we use two different ways to deploy nodes: grid and random uniform distribution. In the grid topology, the number of nodes is 85 and the distance between nodes is 40 m. In the random topology, the number of nodes is 100. The field size of both network topologies is 250 m × 250 m. The traffic type of data sending is called Constant Bit Rate, and half the sensor nodes send data to the gateway. The coordinator node’s energy is infinite, while other nodes’ energy is 5 J. The transmission consumption is 0.0744 W and data-reception consumption is 0.0648 W. To compare LBMR performance with AOMDV performance, the following experiments consist of three evaluation scenarios: load balancing, route maintenance, and delivery ratio. 

### 4.1. Load Balancing

The data gateway is the only node in Layer 0. The nodes, N-hops away from the gateway, are called Layer N. The upper layer is defined as the layer closer to the gateway, and *vice versa.* Layer 1 is the closest layer to the gateway, accumulates all the traffic coming from its lower-layer nodes to the gateway and has the highest load among all the layers. Therefore, upper layers like Layer 1 may become network bottleneck easily. To achieve load-balancing purpose, LEA is developed.

In LEA, we used α to allocate the proportion of estimated load and sample load. In order to find the suitable α, we analyze load balancing of each layer through Flow Variance (FV), which represents each layer’s flow distribution. To calculate FV, Equation (1) first determines the standard deviation (SD) of the layer’s flow. Subsequently, Equation (2) determines FV value. In Equation (1), *N* represents the number of nodes in the layer, *x_i_* represents a specific node’s load of the layer, and $\bar{x}$ represents the average load of the layer. The closer FV is to zero, the more balanced the flow distribution of this layer is:

(1) $$SD=\sqrt{\frac{1}{N}\sum_{i=1}^{N}{\left(x_{i}-\bar{x}\right)}^{2}}$$

(2) $$FV(\%)=\frac{SD}{\bar{x}}\times 100$$

**Figure 5:**
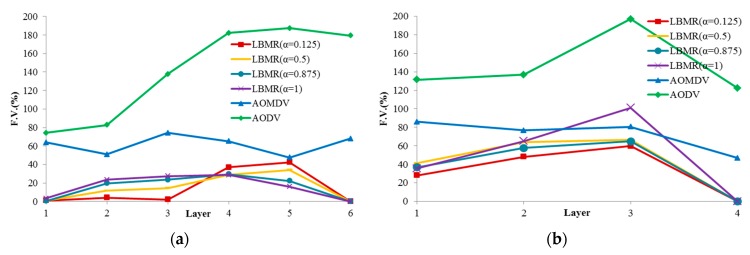
Flow variance of each layer. (**a**) Grid topology; (**b**) Random topology.

In our LBMR design, a good load-balancing mechanism should make FV as close to 0 as possible. More ideally, FV should decrease as the layer number decreases. Figure 5 shows load balancing of each layer based on FV with four different α values (α = 0.125, α = 0.5, α = 0.875, α = 1). From our experiment results, when α is 0.125, LBMR gives the smallest FV in layer 1, layer 2 and layer 3. As mentioned, smaller FV represents better traffic distribution on each layer. In the condition (α = 0.125), FV is smaller in the upper layers compared with the lower layers for both grid and random topologies. This proves LBMR is good for WSN because the upper layers tend to gather more traffic from the lower layers to the gateway, and therefore needs more load-balancing assistance.

Based on FV, LBMR shows much better performance than AOMDV, especially at Layer 1. In grid topology, FV of LBMR with (α = 0.125) is close to 0 at layer 1, 2, and 3. On the contrary, FV of AOMDV is from 50% to 75%. In random topology, FV of AOMDV is over 80% and FV of LBMR can be 30% at layer 1. In AODV scenarios, FV is above 120% in both topologies. AODV is a single-path routing protocol, in which network traffic is aggregated at certain paths and the bottleneck issue becomes more serious. This explains why the load balancing performance of AODV is the worst among three protocols. FV of AODV in both topologies is much higher than LBMR and AOMDV. Therefore, LBMR shows much better load-balancing performance than both AOMDV and AODV do. Moreover, we can observe LBMR of grid topology has better FV performance than that of random topology. This is because the number of connections of grid topology is fixed and the traffic is easier to be predicted and optimized.

**Figure 6:**
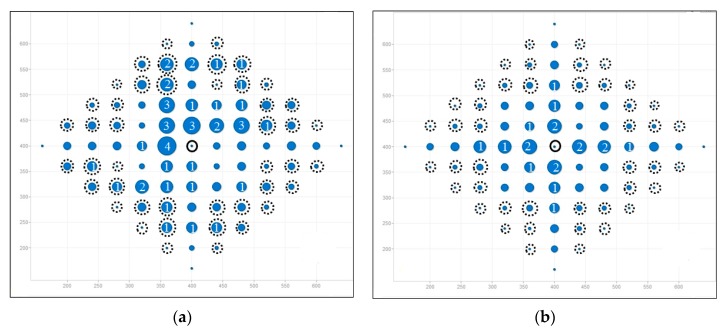
Load distribution in gird topology (total node is 85). (**a**) AOMDV; (**b**) LBMR.

**Figure 7:**
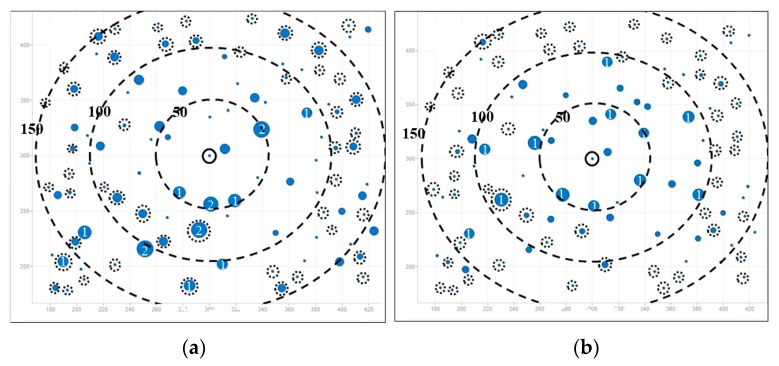
Load distribution in random topology (total node is 100). (**a**) AOMDV; (**b**) LBMR.

From the above analysis of load balancing based on FV benchmark, the performance of AODV is much worse and therefore AODV is proved to with bad-performance in terms of load balancing. From Figure 5, LBMR seems only slightly better than AOMDV. For a further comparison and a more detailed analysis, we visualize the different load-balancing degrees between LBMR and AOMDV in Figure 6 and Figure 7.

Figure 6 and Figure 7 depict load balancing effect in grid and random topologies respectively by using symbols defined in Table 4. The size of a blue circle is proportionally enlarged as N increases. N, representing traffic load, is defined in Table 4 as well. The larger the circles, the more traffic-imbalanced the network is.

Figure 6 shows the comparison of flow distribution at each node in grid topology. Figure 6a shows AOMDV scenario, where large blue circles surround the gateway and it means imbalanced network traffic is aggregated at some nodes, especially at Layer 1. Therefore, bottlenecks are prone to occur at Layer 1. On the contrary, LBMR in Figure 6b is with small circles, which indicates the network traffic is much more balanced. Therefore, LBMR successfully alleviates the network bottleneck issue in AOMDV. For the grid topology shown in Figure 6, Figure 6b shows all layer 1 nodes have the same loading in LBMR but Figure 6a shows half of layer 1 nodes have large loading burdens but the other half have much less burden. 

In Figure 7, three big dashed circles represent three ranges which are 50, 100, and 150 m away from the gateway respectively. Although nodes are randomly deployed in uniform distribution, it is obvious that the number of heavy-loaded nodes in LBMR scenario is relatively less than that in AOMDV, especially nodes nearby the gateway are perfectly load-balanced.

### 4.2. Route Maintenance

**Figure 8:**
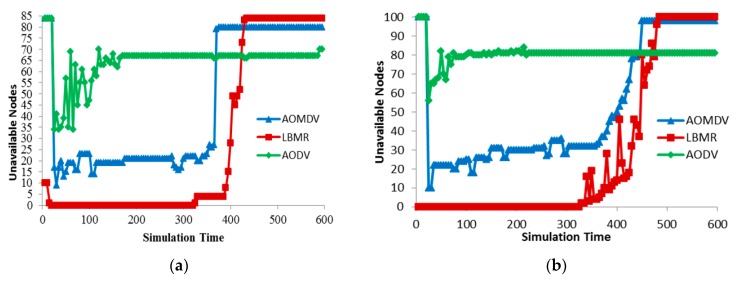
Route maintenance. (**a**) Grid topology (total nodes is 85); (**b**) Random topology (total nodes is 100).

We analyze route maintenance by counting Unreachable Nodes (URNs), which run out of energy or have no routes to the gateway. Since URNs cannot forward data, URNs indicate data-forwarding capability for medical monitoring sensors. Figure 8 shows URNs in three protocols, and LBMR’s URNs are the fewest before most of nodes run out of energy. Because of single next-hop design, nodes in AODV cannot adapt to route failure quickly while encountering large amount of network traffic, and thus URNs increase quickly. URNs in AOMDV are much fewer because of alternative route design. But alternative routes in AOMDV must be disjoint so URNs in AOMDV increases more than 20 quickly. In LBMR, since nodes can quickly choose their best upper-layer nodes to reach the gateway, URNs in LBMR is none most of time, and thus LBMR provides the best performance in terms of route maintenance.

Besides, the initial number of URNs is very high in AODV and AOMDV because nodes require sending RREQ to establish routes to the gateway and this process takes a long time. In LBMR, the route establishing time is very short for nodes within layer 2. For nodes beyond layer 2 in the grid topology, they require a little time to join the network but the process time is much shorter than AODV and AOMDV from Figure 8a. Thus, the route establishing time in LMBR is also shorter than the time in AODV and AOMDV.

Because route maintenance is more challenging in random topology than in grid topology, Figure 8a can clearly illustrate route maintenance effort in three protocols. AODV’s URNs quickly increases quickly while AOMDV’s URNs gradually increase. LBMR’s URNs keeps none in the beginning and remains under 20 after node failure occurs. From Figure 8b, nodes in LBMR rejoin the network quickly, even when URNs keep increasing. LBMR’ URNs remain low until most of the nodes run out of energy in the end. Therefore, LBMR ensures the most nodes participating in medical monitoring services than AODV and AOMDV in the case that nodes have sufficient energy.

### 4.3. Packet Delivery Ratio

**Figure 9:**
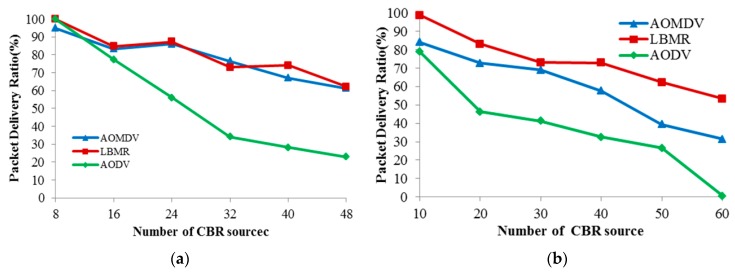
Packet delivery ratio. (**a**) Grid topology; (**b**) Random topology.

Because Packet Delivery Ratio (PDR) directly reflects network performance of medical monitoring applications, PDR is chosen to evaluate routing reliability among the three protocols in Figure 9. While CBR data sources increase, AODV’s PDR quickly drops in both topologies due to worse route maintenance capability, and thus AODV’s reliability is the worst among the three protocols. In random topology, LBMR’s PDR is better than AOMDV’s PDR, and their PDR is quite similar in grid topology. Since network data delivery is more challenging in random topology than grid topology, LBMR is more reliable than AOMDV in the more challenging scenario. Therefore, the load balancing design of LBMR brings more reliable routing service than well-known protocols, AODV and AOMDV, for medical monitoring applications.

## 5. Conclusions

As medical applications grow rapidly, reliable routing is highly desirable to allow medical sensors to accurately deliver data packets to the gateway through multi-hop transmissions. For preventing bottleneck issues in Zigbee’s AODV routing services, LBMR is proposed to provide a load balancing, robust and reliable routing service for medical applications. To achieve these goals, LBMR consists of LRC, LEA, and RM. LRC provides a multipath layer routing service towards the gateway for medical applications, and LEA estimates load information for data senders choosing the upper layer next hop with the least network loading. The synergy of LRC and LEA eliminates the bottlenecks and thus provides a load balancing routing service. In addition, RM detects and recovers route failures to reinforce route robustness, and therefore reliability is accomplished. The experiment results demonstrate that LBMR achieves much better load balancing than AODV and AOMDV according to FV values. Based on URNs, LBMR provides more robust routing than AODV and AOMDV. Furthermore, LBMR’s PDR is better than AODV and AOMDV, and thus LBMR’s data delivery is the most reliable. In conclusion, based on the load balancing design, LBMR provides the most reliable and robust routing service for medical applications compared with the current famous in-use routing solutions, Zigbee’ AODV and its improved multipath version, AOMDV.

## Figures and Tables

**Table 1:** Route Construct Algorithm.

// y is sender and x is receiver. Receive Route Construct; If (${\text{layer}}_{y}{>(\text{layer}}_{x}\;-1$) ) { // Condition 1 avoids routing loop Drop Route Construct; } Else if (${\text{layer}}_{y}{<(\text{layer}}_{x}\;-1$)) { // Condition 2 ensures the shortest path. If (routing table is empty) { Add y node to routing table; } Else { Delete all routing entry & Add y node to Routing Table; } ${\text{layer}}_{x}{=\text{layer}}_{y}+1$; For the new Route Construct layer = ${\text{layer}}_{x}$; source = x; Broadcast the new Route Construct; } Else if (${\text{layer}}_{y}{=(\text{layer}}_{x}-1$)) { // Condition 3 makes nodes build multi-paths. Add y node to Routing Table; }

**Table 2:** Load Estimation Algorithm (LEA).

${\text{Estimated}\_\text{Load}}_{0}=\text{SampleLoad}$ // For time slot i, If(SampleLoad==0) { ${\text{Estimated}\_\text{Load}}_{i}{=\text{Estimated}\_\text{Load}}_{i-1}/2$; } Else { ${\text{Estimated}\_\text{Load}}_{i}=$ $(1-\alpha )*{\text{Estimated}\_\text{Load}}_{i-1}+\alpha \times \text{SampleLoad}$; }

**Table 3:** Environment parameters in ns2.

Parameter	Value
Simulation time	600 s
Traffic type	Constant Bit Rate
Transmission range	50 m
Phy/MAC layer	IEEE 802.15.4
Packet size	100 Bytes
Number of nodes	85 and 100
Data Sending Interval	1 s

**Table 4:** Symbol description.

Symbol	Description
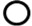	data gateway
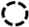	CBR source
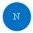	Node with traffic load above 10% of total source data traffic, where, N = (Node’s load/Total source data traffic) × 10. N is 1, when the load is from 10% to 19%; N is 2, when the load is from 20% to 29%, and so on.
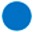	Node with traffic load under 10% of total source data traffic
